# Adaora A. Adimora, in memoriam

**DOI:** 10.1002/jia2.26210

**Published:** 2024-01-28

**Authors:** Judith D. Auerbach

**Affiliations:** ^1^ Department of Medicine University of California San Francisco San Francisco California USA

The global HIV community has lost a true champion with the passing of Dr. Adaora Alise Adimora on 1 January 2024. Ada was an extraordinary woman, scholar, clinician, mentor, advocate and dear friend who served as the Sarah Graham Kenan Distinguished Professor of Medicine at The University of North Carolina (UNC) at Chapel Hill, United States. She was devoted to elevating attention to women in the HIV research and policy agenda, and to addressing pervasive racial and gender inequalities in HIV and associated healthcare. Ada was a beloved clinician who provided tireless and compassionate care to all her patients, and she was a mentor to many early career investigators and healthcare providers in the United States and globally.

As a physician‐epidemiologist, Ada approached infectious disease risks, lived experiences and outcomes in the context of the social forces and structural factors that influenced them. She applied this bio‐social worldview in all of her work, forcing colleagues to confront and address uncomfortable social realities, such as racial discrimination, in health research and care and cajoling many of us across diverse disciplines—as only she could—to co‐author papers to advance attention to these important issues [1, 2].

Ada's scholarship helped characterize the epidemiology of HIV and other sexually transmitted infections, with special attention to the prevention and treatment of HIV among Black women in the United States and globally. Her seminal work highlighted the role of sexual network patterns in the spread of HIV, elucidating the importance of macroeconomic and social forces in racial disparities in the U.S. HIV epidemic. Her later work focused on the role of incarceration in the epidemiology of HIV among Black Americans, demonstrated the increased risk of comorbidities and mortality among women with and at risk for HIV, and highlighted the complex role of structural factors, such as poverty, racism, and insufficient health insurance, in health outcomes. .

Ada was a renowned advocate and leader in HIV and women's health. She served as the Chair of the U.S. National Institutes of Health (NIH) HIV Prevention Trials Network Women at Risk Committee, was a leader of the NIH‐funded Multicenter AIDS Cohort/Women's Interagency HIV Study Combined Cohort Study and served on The Well Project's Women's Research Initiative on HIV/AIDS, among other commitments aimed at ensuring that the voices and experiences of women were always a part of the national and global HIV prevention, care and research agenda.

Ada had a particular passion for redressing the problem that data from U.S. women were not available to inform guidelines for their use of pre‐exposure prophylaxis (PrEP). Even among Black women, who bear the highest burden of HIV among women in the United States, the overall incidence is too low for conducting a randomized controlled trial (RCT) with HIV incidence as its outcome, given the very large sample size requirements for such an RCT. As such, Ada worked tirelessly to propose alternative strategies for obtaining evidence of PrEP efficacy, such as evaluating relationships between HIV incidence and drug concentrations measured among participants in RCTs conducted in high‐incidence settings and then applying these observations to drug concentrations measured among at‐risk women in lower‐incidence settings [3]. In this way and others, Ada never allowed “accepted wisdom” to limit her view of what was possible when an issue of equity was at stake.

In recognition of her standing in the field, Ada was invited to serve as a member of several high‐level bodies, including the Presidential Advisory Committee on HIV/AIDS, the NIH Office of AIDS Research Advisory Council, the U.S. Department of Health and Human Services Antiretroviral Treatment Guidelines Panel and the U.S. Centers for Disease Control and Prevention Advisory Committee to the Director. She served two terms as a North American representative on the International AIDS Society Governing Council, and was elected in 2019 to the National Academy of Medicine. In all these positions, Ada brought determination, boldness and righteousness to the table; she was referred to by the editors of the “POZ Magazine 100” as one of the “bravest, most dogged and downright effective AIDS fighters we know.”

Ada was an unparalleled and selfless mentor to numerous students, fellows, research staff and junior colleagues—at UNC and also in Malawi, China and Cameroon—helping them launch and succeed in clinical practice and research careers. She received an NIH training grant focused on increasing the pool of HIV researchers, especially African American investigators, served as the Principal Investigator of the UNC Fogarty AIDS International Training and Research Program from its inception in 1998 to its end in 2014, and of UNC's Sexually Transmitted Infections/HIV Training Program. As a mentor, Ada's seriousness and no‐nonsense approach could be intimidating; but she had a way of putting people at ease with a basic human connection. One early career investigator recalled, “I remember the first time I met her at a Women's Interagency HIV Study meeting. I felt like such a groupie, nervous to introduce myself. But she was so down to earth; instead of babbling on about my [early career NIH grant], we talked about Black hair. That is a memory I will always cherish.”
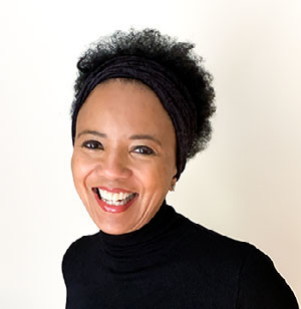


Photo credit: Bria Adimora Godley


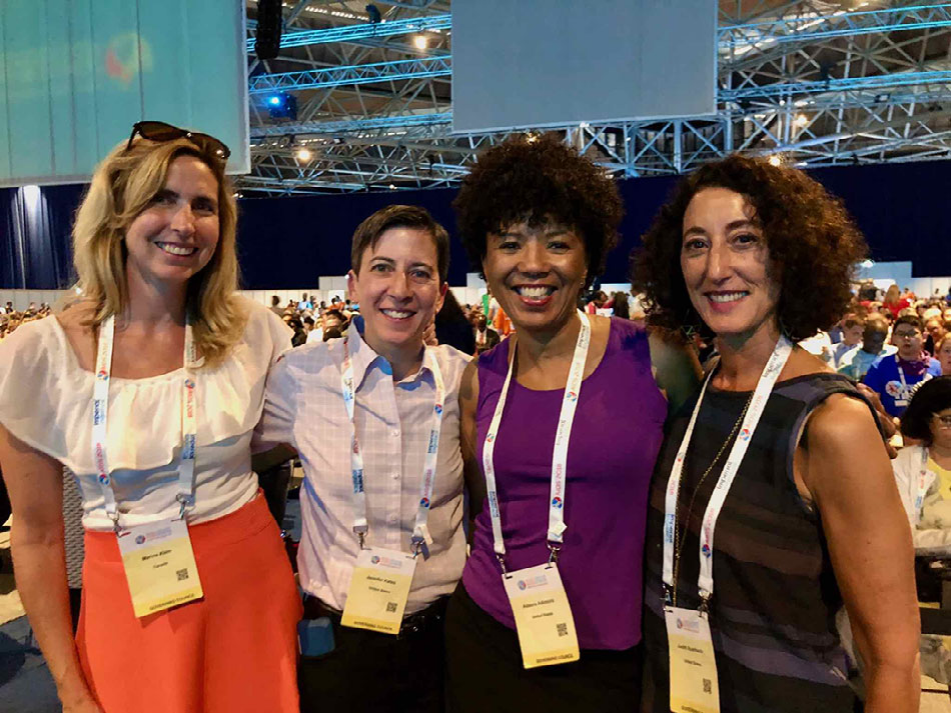
 From left: Marina Klein, Jennifer Kates, Ada Adimora and Judith Auerbach at AIDS 2018, Amsterdam

In addition to her outstanding contributions to the field, Ada was considered by all who knew her as beautiful, brilliant, radiant, passionate, feisty, funny, loving and generous. She was fastidious about both her work and her appearance, which is why many people did not even know she had been ill. Those of us who did know were shocked to find Ada reviewing abstracts for the Conference on Retroviruses and Opportunistic Infections only weeks before she died; but she was determined not to be seen as shirking her responsibilities.

Ada did not suffer fools easily, but she always listened with an open mind and an interest in expanding her understanding. She was devoted to her family and her community of friends, who collectively feel her loss profoundly and are committed to carrying forward her legacy in the fight for health equity, social justice and gender equality.

## COMPETING INTERESTS

The author declares no competing interests.

## AUTHOR CONTRIBUTIONS

JDA conceptualized and wrote the manuscript.

## Data Availability

Data sharing not applicable to this article as no datasets were generated or analysed during the current study.
